# Navigating Performance Standards for Face Mask Materials: A Custom‐Built Apparatus for Measuring Particle Filtration Efficiency

**DOI:** 10.1002/gch2.202100052

**Published:** 2021-08-05

**Authors:** Ryan J. LaRue, Patrick Morkus, Scott Laengert, Sarah Rassenberg, Mohamad Amin Halali, John W. Colenbrander, Catherine M. Clase, David R. Latulippe, Charles‐François de Lannoy

**Affiliations:** ^1^ Centre of Excellence in Protective Equipment and Materials (CEPEM) 1280 Main St. W. Hamilton ON Canada; ^2^ McMaster University Department of Chemical Engineering Canada; ^3^ McMaster University Department of Mechanical Engineering Canada; ^4^ McMaster University Department of Medicine Canada; ^5^ McMaster University Department of Health Research Methods Evidence and Impact Canada; ^6^ St. Joseph's Healthcare Hamilton Canada

**Keywords:** aerosols, COVID‐19, face masks, particle filtration efficiency, standards

## Abstract

Public health agencies have recommended the community use of face masks to reduce the transmission of airborne diseases like COVID‐19. Virus transmission is reduced when masks act as efficient filters, thus evaluating mask particle filtration efficiency (PFE) is essential. However, the high cost and long lead times associated with purchasing turn‐key PFE systems or hiring certified laboratories hampers the testing of filter materials. There is a clear need for “custom” PFE test systems; however, the variety of standards that prescribe (medical) face mask PFE testing (e.g., ASTM International, NIOSH) vary widely in their protocols and clarity of guidelines. Herein, the development is described of an “in‐house” PFE system and method for testing face masks in the context of current standards for medical masks. Pursuant to the ASTM International standards, the system uses an aerosol of latex spheres (0.1 µm nominal size) with particle concentrations upstream and downstream of the mask material measured using a laser particle analyzer. PFE measurements are obtained for a variety of common fabrics and medical masks. The approach described in this work conforms to the current standards for PFE testing while providing the flexibility to adapt to changing needs and filtration conditions.

## Graphical abstract

Non‐medical face masks are a key tool for preventing the spread of airborne disease during a pandemic. The development and evaluation are presented of a new apparatus for measuring the particle filtration efficiency of these masks in the context of major prevailing performance standards. This custom apparatus enables innovation in filter materials by efficiently and cost‐effectively measuring filtration performance.

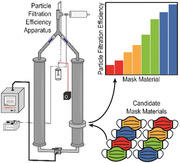

## 1 Introduction

Public health agencies recommend face masks for the general population to limit the spread of COVID‐19 and other droplet‐ and aerosol‐transmitted diseases.^[^ ^1^ ^]^ Masks mandates have been effective in reducing transmission,^[^ ^2^ ^]^ suggesting that untested community masks provide useful filtration. In fact, modelling studies suggest that COVID‐19 transmission decreases nearly proportionally to the combined product of mask effectiveness and adoption rate, and that these are synergistic with other population‐based measures in reducing hospitalizations and deaths.^[^ ^3^ ^]^

The numbers of certified medical masks and respirators needed by health care and other frontline workers has risen dramatically, challenging existing manufacture and supply chains, and leading to the need for rapid testing and certification of new materials for new manufacturers. Standardized methods from organizations such as ASTM International and the US National Institute for Occupational Safety and Health (NIOSH) exist for testing medical masks; however, the details of these methods vary widely and each organization has established its own performance criteria.

Particle filtration efficiency (PFE) is the most important property of face masks as it relates to their ability to filter small particles such as aerosols. Medical masks must achieve specific PFE targets^[^ ^4^, ^5^, ^6^ ^]^ in order to be certified by governing bodies such as ASTM International or NIOSH. Surgical masks are certified by ASTM, while N95 respirators are certified by NIOSH, but both masks must pass specific PFE cutoffs. For example, N95 masks must achieve 95% filtration of aerosols composed of salt particles with a count‐mean diameter of 0.075 µm, while ASTM 2100 L3 surgical masks must achieve 98% filtration of aerosols composed of latex spheres with a mean diameter of 0.1 µm.

Prior to the COVID‐19 pandemic, there were no methods for testing non‐medical face masks to be used in lower‐risk community settings, such as those used by the general public. Only recently, ASTM International developed a framework for the design, performance evaluation, and labelling of general use face masks (termed “barrier face coverings”).^[^ ^7^ ^]^ As such, previous research on the PFE of non‐medical face masks for community use, and common materials used to make them, have used disparate methods making it challenging to compare the results from those studies.^[^ ^8^, ^9^, ^10^ ^]^ It is unlikely that non‐medical face masks made of textiles will approach the standards associated with medical masks, and we do not yet know what constitutes an acceptable PFE for a non‐medical mask or which is the most important particle size or type to study. As the debate continues, standardized testing protocols among research labs are critical to help identify useful non‐medical masks and mask materials. Furthermore, standard testing protocols will help determine achievable PFE values for non‐medical masks and guide the creation of reasonable standards for non‐medical face masks. There are three standard avenues for determining the PFE value of a face mask material:

1. Contract the services of a certification laboratory (e.g., Nelson Laboratory [Utah, USA] or Kinectrics Inc. [Ontario, Canada]);
2. Purchase a turn‐key filtration efficiency apparatus (e.g., TSI Automated Filter Tester 8130A); or
3. Design and build a custom filtration testing apparatus.

The first two options are expensive (>$1000 per tested sample and estimated at >$150 000 for a designated apparatus) and during the COVID‐19 pandemic, there have been delays due to long lead times and supply issues. The high cost and limited access to PFE testing—in conjunction with a lack of coherent guidance on standardized performance evaluation—has resulted in researchers using various custom built test systems, which generally are based on one or more of the standards for certifying medical masks.

The application of mask material standards varies by product (i.e., respirators versus surgical‐type masks versus community‐use barrier face covering, etc.) and jurisdiction. In the literature and in the published standards, testing apparatuses vary widely by their test conditions. Standards vary by parameters including the particle chemistry (e.g., latex,^[^ ^4^, ^5^ ^]^ salt^[^ ^6^, ^7^ ^]^), size (e.g., 0.1 µm,^[^ ^4^ ^]^ 0.1–5 µm,^[^ ^5^ ^]^ 0.075 ± 0.020 µm^[^ ^6^, ^7^ ^]^), particle concentration (e.g., 10–100 particles cm^–3^ and <100 particles cm^–3^,^[^ ^4^ ^]^ <200 mg NaCl m^–3[^ ^6^ ^]^), air flow rate (e.g., 1–1000 L min^–1^,^[^ ^4^ ^]^ 85 ± 4 L min^–1,[^ ^6^, ^7^ ^]^), face velocity at the sample (e.g., 0–25 cm s^–1^,^[^ ^4^ ^]^ unspecified,^[^ ^6^ ^]^ 10 cm s^–1[^ ^7^ ^]^), and temperature/humidity.^[^ ^4^, ^5^, ^6^, ^7^ ^]^ Thus, navigating these various test conditions can prove to be confusing for researchers. As such, we present a brief comparison of the four major standards that have been used to date; for a more detailed discussion of each of these standards, we refer the reader to Section S1 (Supporting Information) as well as Table 2. Also in the Supporting Information, we present brief details on proposed standards that have been published by three other organizations: the American Association of Textile Chemists and Colorists (AATCC),^[^ ^11^ ^]^ the Association Française de Normalisation (AFNOR) from France^[^ ^12^ ^]^ and the Bureau de Normalisation (BNQ) from Quebec.^[^ ^13^ ^]^ The three main standards are as follows:

1. “NIOSH Procedure TEB‐APR‐STP‐0059: Determination of Particulate Filter Efficiency for N95 Series Filters Against Solid Particulates for Non‐Powered, Air‐Purifying Respirators Standard Testing Procedure (STP)” (Revision 3.2, December 13^th^, 2019):
  - Designed for the certification of N95 candidate respirators, but not medical or non‐medical face masks that do not seal to the face.^[^ ^6^ ^]^ It mandates the use of a TSI Automated Filter Tester Model 8130 or 8130A system (or equivalent) built by TSI Incorporated (§3.1.1). All equipment including aerosol generation, neutralization, and concentration‐measuring is incorporated into the instrument.
  - Aerosol particles are to be produced from a 2% solution of sodium chloride in distilled water (§3.1.5), providing a concentration of aerosol particles in the range of 12–25 mg m^–3^, under the upper limit of particle concentration (200 mg m^–3^) set in §5.1. The particle size distribution is to have a “count median diameter of 0.075 ± 0.020 micrometer and a geometric standard deviation not exceeding 1.86′′ (§5.1). The final dry particle to be filtered by the mask material is smaller by NIOSH standards (0.075 µm) than that stipulated by ASTM (0.1 µm). The aerosolized particles are subsequently “neutralized to the Boltzmann equilibrium state” (§5.1) via a high‐voltage aerosol neutralizer (§4.2.4).
  - The mixture of air and aerosolized particles must have a temperature and humidity of 25 ± 5 °C and 30 ± 10%, respectively.
  - The total flow rate reaching the sample is variable depending on the construction of the mask. For a single‐filter mask (e.g., a typical N95 respirator with area ≈ 150 cm^2^), a flow rate of 85 ± 4 L min^–1^ is required corresponding to a face velocity of roughly 10 cm s^–1^.
  - The penetration (i.e., downstream concentration divided by the upstream concentration) is reported by the instrument and filtration efficiency can be obtained via Equation (1)
(1) $$\begin{array}{c} \text{efficiency}\;\;=\;\;\left[1-\text{penetration}\right]\;\;\times \;\;100\% \end{array}$$
2. “ASTM F2100‐19e1: Standard Specification for Performance of Materials Used in Medical Face Masks” (approved in 2001, latest update 2019) in conjunction with “ASTM F2299/F2299M‐03(2017): Standard Test Method for Determining the Initial Efficiency of Materials Used in Medical Face Masks to Penetration by Particulates Using Latex Spheres” (approved in 2003, latest updated 2017)
  - ASTM F2100 provides an overview of the performance of a mask material using five tests to evaluate different performance characteristics, one of which is the “sub‐micrometer particulate filtration efficiency test” which is measured using 0.1 micrometer particles (§6.1). ASTM F2100 references ASTM F2299 for the methodology by which to conduct a PFE measurement and assigns “level” based on how they perform in all of the tests, combined. Level 1 masks exhibit a sub‐micrometer PFE of ≥95 percent, and Levels 2 and 3 masks exhibit a sub‐micrometer PFE of ≥98 percent.
  - ASTM F2299 seeks to “establish procedures for measuring the initial particle filtration efficiency of materials used in medical face masks using monodispersed aerosols […] in the size range 0.1 µm to 5.0 µm2.′′^[^ ^4^ ^]^ Unlike the NIOSH standard, there is no prescribed apparatus in ASTM F2299.
  - The aerosol particles are defined to be “monodisperse latex spheres” (§3.1.3.1; §4.1) with a “diameter of 0.1 to 5 µm” at a concentration of “less than 10^2^ particles/cm^3^′′ (§3.1.1.1). No further composition details (such as particle chemical composition and surface chemistry/charge) are specified. Note that while ASTM F2299 details how PFE can be performed using particles between 0.1 and 5 µm, ASTM F2100 specifies that medical facemasks must be tested with 0.1 µm particles. The aerosols are to be atomized from “a dilute latex/deionized water suspension (§7.2.2) via a generator which must output 10^7^ to 10^8^ particles/m^3^” (10–100 particles/cm^3^; §7.2.1). The standard specifically recommends the use of a Collison‐type atomizer or another similar atomizer (§7.2.2). Subsequently, surface charge on the aerosol is to be neutralized prior to injection into the test system with a “typical ionizing flux of 10^3^ mCi m^−3^ s^−1^” (§7.3) such as that provided by a ^85^Kr, ^210^Po, or corona discharge source.
  - ASTM F2299 specifies many parameters (or acceptable ranges) of the test chamber, such as the chamber length/diameter and sampling geometries, as opposed to the NIOSH standard. For a detailed discussion of these design parameters, refer to §S1 in the Supporting Information.
  - The mixture of air and aerosol particles that is fed to the preconditioned material sample must have a relative humidity range of 30 to 50%, held within ± 5% during a given test (§7.4), but there is no temperature prescription.
  - The flow rates and face velocities of the air/aerosol fall within the range of 0.035 to 35 cubic feet per minute (1 L min^–1^ to 1 m^3^ min^–1^), and the corresponding face velocity at the sample must be between 1 to 50 ft min^–1^ (0.5–25 cm s^–1^). Note the wide range of acceptable face velocities and air flow rates through the sample, which have 50‐fold and 1000‐fold ranges, respectively.
  - Particle concentrations are to be measured using isokinetic sampling probes (before and after the material sample) leading to a calibrated “automatic, single particle light‐scattering counter” (§7.9.1). The efficiency by which the material rejects the aerosol particles is calculated as follows (§10.4.6)
(2) $$\begin{array}{c} \text{efficiency}\;=\left[1-\frac{\text{average}\;\text{downstream}\;\text{particle}\;\text{concentration}}{\text{average}\;\text{upstream}\;\text{particle}\;\text{concentration}}\right]\;\times 100\% \end{array}$$
3. The new “ASTM F3502‐21: Standard Specification for Barrier Face Coverings” standard (last updated February 15th 2021)
  - Was created in response to the need for standardized masks for the general public to wear during the ongoing COVID‐19 pandemic.^[^ ^7^ ^]^ Unlike previous standards, it seeks to issue guidelines for generic face coverings (not just “medical masks”) with specifications including “design criteria, […] labelling, and user information” (§1.2.5) in addition to the traditional performance evaluation and test methods. Design features, sub‐micrometer particle filtration efficiency, breathability, leakage and fit, and considerations if the mask is reusable/washable are all prescribed. The filtration performance benchmarks under ASTM F3502 are much more demanding: “Level 1” certified masks will have sub‐micrometer PFEs measured at 20% or more which increases to 50% or more for “Level 2” certified masks. ASTM notably supplants their own PFE testing procedure in F2299 in favor of NIOSH's TEB‐APR‐STP‐0059 test procedure, and therefore the TSI 8130/8130A system.
  - The aerosol particles are designated as “polydisperse sodium chloride (NaCl) aerosols with a count median diameter of 75 ± 20 nm electrical mobile diameter and a geometric standard deviation of ≤1.86 to give a mass median aerodynamic diameter of 0.3 µm” (§4.1.1).
  - The particle concentrations are to be “determined by using a forward‐light‐scattering photometer or equivalent” (§8.1.3.3) like in the NIOSH standard, and PFE is to be calculated according to Equation (1), as before.
  - Samples should be supported by a mesh screen (70% or more open area), a wire frame, or another such device to support samples that lay flat against the filter holder to “prevent [the] collapse of the product into the equipment, which can potentially affect test results” (§8.1.2.1)
  - The flow rates are specified as 85 ± 4 [L min^–1^ of air] (§8.1.3.5), but if the sample lays flat in the filter holder, then the flow rates must be adjusted to achieve a face velocity of 10 ± 0.5 cm s^−1^.

The ad hoc mask material testing apparatuses found in the existing literature generally approximate the NIOSH or ASTM F2100/F2299 standards presented above. However, the researchers have had the opportunity to choose or vary design or operational parameters to their liking. For example, variations in face velocity at the sample, air/aerosol flow rate, sample size (area), and aerosol particle compositions have been used. Many recent studies have evaluated mask materials using custom‐built apparatuses that utilize a sodium chloride aerosol, approximating the NIOSH standard. For example, Rogak et al. (2020), Zangmeister et al. (2020), Drewnick et al. (2020), and Joo et al. (2021) all built apparatuses that generated a sodium chloride aerosol (of various sizes) which was charge‐neutralized, diluted with filtered air and sent to the material sample, where particle concentration measurements were taken with various combinations of optical particle sizers, condensation particle counters, and/or scanning mobility particle sizers/differential mobility analyzers.^[^ ^9^, ^14^, ^15^, ^16^ ^]^ Konda et al. (2020) and Hao et al. (2020) built similar apparatuses but did not include the charge neutralizer.^[^ ^8^, ^17^ ^]^ In these studies, the air flow rate at the sample varied (sometimes to probe flow rate/velocity effects) between 1 and 90 L min^–1^; however, face velocities fell between 5.3 and 25 cm s^–1^. Sample sizes appear to vary between ≈3.4 and 59 cm^2^.

Conversely, fewer studies have evaluated mask materials via apparatuses that utilize a latex aerosol, approximating the ASTM F2100/F2299 standards. For example, Bagheri et al. (2021), Shakya et al. (2016), and Lu et al. (2020) constructed apparatuses that generate a polystyrene latex aerosol which was diluted and sent to the material sample, where particle concentration measurements were performed using various particle analyzers or scanning mobility particle sizers.^[^ ^18^, ^19^, ^20^ ^]^ Whereas Lu et al. utilized a charge neutralizer downstream of their aerosol generator, the authors of the other two studies did not. Air flow rates at the sample also varied slightly—but within the constraints of the F2299 standard—from ≈7.3 to 19 L min^–1^. The air face velocities were 2 and 10 cm s^–1^ (within the standard's range) for the studies by Bagheri et al. and Lu et al., respectively, while Shakya et al. did not clearly state their face velocity.^[^ ^18^, ^19^, ^20^ ^]^ Furthermore, the authors tested various sizes of latex spheres (i.e., overall, 20 nm up to 2500 nm) with both Shakya et al. and Lu et al. using the prescribed 100 nm (0.1 µm) particle size in at least some of their tests.

In this work, we describe the challenges we faced in creating a PFE apparatus which conforms as closely as possible to the existing ASTM F2100/F2299 standards. Of the main prevailing standards (i.e., NIOSH and ASTM F2100/F2299), the ASTM standard offers greater flexibility in parameters which can be varied (e.g., air flow rate) to study filtration performance that may affect the PFE in non‐medical face masks. However, as we demonstrate, this flexibility provides additional levels of complication when designing such an apparatus.

## 2 Experimental Section

### 2.1 Chemicals and Materials Tested

Chemicals were purchased from Sigma‐Aldrich and used as received. Styrene monomer (≥99%) was purified by passing it through a glass column packed with aluminum oxide inhibitor remover designed to remove *tert*‐butylcatechol. Deionized water (≈0.037 µS cm^–1^) was obtained from a Sartorius Arium water purification system. 

A woven 100% cotton muslin fabric (Muslin CT) with a nominal weight of 147 g m^–2^ was obtained from Veratex Lining Ltd., QC and a bamboo/spandex blend from D. Zinman Textiles, QC. Other candidate mask materials were obtained from a local fabric retailer (Fabricland). These materials include two different 100% cotton woven fabrics (with different prints), a cotton/spandex knitted fabric, two cotton/polyester knitted fabrics (one “generic” and one “sweater fabric”), and a nonwoven cotton/polypropylene blend batting material. A summary of known fabric properties is shown in **Table** **1**. To benchmark the new apparatus, certified medical masks were also obtained from a local hospital including ASTM 2100 Level 2 (L2) and Level 3 (L3; Halyard) certified medical masks as well as an N95 respirator (3M).

**Table 1:** List of candidate fabric materials for this study. “TPI” represents the thread count of a woven fabric in threads per inch

Candidate material identifier	Composition	Weight [g m^–2^]	Construction
Veratex Muslin CT	100% cotton muslin	150	Woven, 118 TPI
Fabric Store Cotton A	100% cotton	111	Woven, 200 TPI
Fabric Store Cotton B	100% cotton	163	Woven, 124 TPI
D. Zinman Bamboo/Spandex Blend	92%/8% bamboo/spandex	253	Knit
Fabric Store Cotton/Spandex Blend	94%/6% cotton/spandex	302	Knit
Fabric Store Cotton/Polyester Blend	70%/30% cotton/polyester	228	Knit
Fabric Store Sweater Fabric Blend	51%/49% cotton/polyester	254	Knit with Nap
Fabric Store Batting Material Blend	87.5%/12.5% cotton/polypropylene	136	Nonwoven

Circular samples of approximately 85 mm diameter were cut from each material to be tested; no further modifications (e.g., washing) were made to the materials. The fabric circles were clamped into the sample holder of the PFE apparatus for testing. The actual diameter of the sample contacting the airflow was 73 mm, with the remaining material used to tightly secure the sample. For assembled masks, the side that contacts the face was oriented away from the aerosol fed to the material.

### 2.2 Synthesis and Characterization of Latex Spheres

Monodisperse anionic polystyrene latex spheres were synthesized via emulsion polymerization. The reaction was operated in a monomer‐starved semibatch mode according to the procedures described in previous studies.^[^ ^21^, ^22^ ^]^ A 250 mL three‐neck round bottom flask was charged with deionized water (160 mL) and heated to 70 °C in a stirred oil bath. The flask was subsequently purged with nitrogen and inhibitor‐free styrene monomer (2.1 mL) was added to the purged, stirred flask. After 10 min at 70 °C, sodium dodecyl sulfate (0.235 g) dissolved in deionized water (8 mL) was added. After another 5 min, potassium persulfate (0.5 g) dissolved in deionized water (2 mL) was added. Over the next 5 h, additional inhibitor‐free styrene (20 mL) was slowly infused into the flask at 66 µL min^–1^ using a syringe pump. The reaction proceeded for an additional 17 h after the styrene infusion finished. The flask was then opened and left to cool to end the polymerization. The synthesized polystyrene latex emulsion was dialyzed against deionized water for five days in SnakeSkin dialysis tubing (3500 Da molecular weight cutoff) with the deionized water replaced daily. The emulsion was removed from the dialysis tubing and stored in the refrigerator at 4 °C until it was used.

Dynamic light scattering (DLS) was performed using a Brookhaven 90Plus analyzer with a 659 nm laser and detector angle of 90°. The data was analyzed using the built‐in Particle Solutions Software (v2.6; Brookhaven Instruments Corporation). The latex suspension was diluted with deionized water until the particle count was approximately 500 kilo‐counts per second (kcps). It was determined that the particle diameter was 125 ± 3 nm with a reported polydispersity of 0.289 ± 0.006.

Zeta potential measurements were obtained using a ZetaPlus zeta potential analyzer (Brookhaven Instruments Corp.) in phase analysis light scattering mode. Samples were prepared by adding aliquots of latex to a 5 × 10^−3^ m NaCl solution, again diluting the latex suspension to achieve an approximate particle count of 500 kcps. Five replicate measurements (each consisting of 30 runs) were perfomed, yielding zeta potential values of −55.1 ± 2.8 mV, where the error represents the standard deviation about the average of the five replicates. These measurements indicate that the particles hold a negative charge and form a stable suspension. DLS and zeta potential data can be found in Tables S2 and S3 in the Supporting Information.

### 2.3 Particle Filtration Efficiency Apparatus

**Figure 1:**
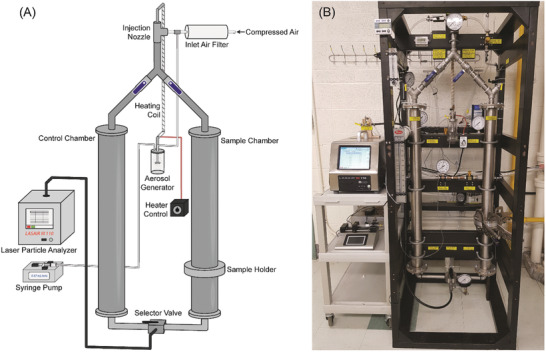
A) Simplified illustration and B) photograph of the McMaster PFE apparatus. A full piping and instrumentation diagram is show in Figure S1 and Table S1 in the Supporting Information.

We constructed equipment in keeping with the standards of ASTM International, as described below and illustrated in **Figure** **1**. A single‐jet Blaustein Atomizing Module (BLAM; CHTech) aerosol generator was used to produce an aerosol containing latex spheres. A stream of filtered air (obtained by passing through GE Healthcare Whatman 0.3 µm HEPA‐CAP and 0.2 µm POLYCAP TF filters in series) entered the aerosol generator at 20 psi (6.9 kPa) and aerosolized a fraction of a 5 mg L^–1^ suspension of latex spheres injected into the device via a syringe pump (KD Scientific Model 100). The aerosolized wet particles were dried by passing the air stream exiting the aerosol generator through a tubular heat exchanger consisting of 5/8′′ stainless steel tubing wrapped with eight feet of heating coil having a power output of 216 W (BriskHeat). The heater output was set to 40% of the device maximum (≈86 W) according to its adjustable dial; this produced an average outer wall temperature of 112 °C (with a standard deviation of ≈1 °C) as measured by a surface‐mounted thermocouple (Taylor USA). Heater performance is summarized in Figure S4 in the Supporting Information.

The dried aerosolized particles were then mixed with a larger volume of filtered air to achieve a total air flow rate of 28.3 L min^–1^ (i.e., 1 cubic foot per minute). This value was chosen because it is the exact flow rate sampled by the laser particle analyzer instrument downstream in the system. The air stream carrying the latex particles was sent to either one of two identical vertical chambers (i.e., smooth‐walled stainless‐steel pipes): a “control” chamber containing no mask material, or a “sample” chamber into which a circular cut‐out of fabric is inserted using a removable sample holder. The chambers both have an inner diameter of 73 mm, matching that of the sample holder. The sample holder uses a grooved ring and recessed bolts to tightly seal in a mask material, and subsequently, the removable holder is inserted into the gap in the sample chamber and is tightly sealed into the apparatus with rubber gaskets and clamps (Figure S2, Supporting Information).

The diameter of the fabric sample which contacts the air stream is 73 mm (area = 41.9 cm^2^); it was sealed within the sample chamber for the duration of a test. The air stream leaving either the “control” or “sample” chambers was diverted to a laser particle analyzer (Particle Measuring Systems LASAIR III 110) to measure the number concentration of latex particles. The particle analyzer has stated lower and upper limits for particle concentrations of 2 × 10^–4^ and ≈34 particles per cm^3^ of air (7 and ≈950 000 particles per cubic foot), respectively. For measurements of the latex particle concentrations, particle concentrations were reported within a “bin” having lower and upper limits of 0.10–0.15 µm, corresponding to the approximate size of singlet latex particles in the aerosol. However, other bin sizes can be used, and multiple bins can be evaluated simultaneously up to a particle size of 5 µm.

Additional provisions are included in the apparatus, such as equipment to flush the chambers and particle analyzer with clean, filtered air, and the requisite valving and instrumentation (Figure 1). A full piping and instrumentation diagram is shown in Figure S1 and Table S1 in the Supporting Information.

### 2.4 Particle Filtration Efficiency Experiments

During experiments, latex suspension was infused into the aerosol generator at flow rates ranging from ≈60 to 100 µL min^–1^ to maintain a consistent particle output in the approximate range of 14–25 particles per cm^3^ (400 000–700 000 particles per cubic foot) in the 0.10–0.15 µm size bin. This range of flow rates was needed because variations were observed in latex particle concentrations downstream of the aerosol generator, likely attributable to variations in the amount of latex suspension which was caught by the aerosol generator's liquid trap.

To measure the PFE of a given fabric sample, first the latex particle aerosol was diverted through the control chamber and then directed to the particle analyzer. Three consecutive measurements of particle concentrations were taken in rapid succession, each lasting one minute in duration. The particle analyzer reports the time‐averaged concentration of particles during the analysis period, that is, the average concentration of particles in the one‐minute (28.3 L) sample. Following these baseline measurements to establish a steady particle number and gas flow rate, the aerosol was diverted into the sample chamber. Once the system reached equilibrium (typically 60‐90 seconds), three more consecutive one‐minute measurements were taken in rapid succession. These sample measurements represent the concentration of particles that pass through the fabric sample. Subsequently, another three particle concentration measurements were taken from the control chamber by diverting the aerosol stream back into the control chamber, to verify that upstream particle concentration had not materially changed throughout the evaluation of the sample. As the two chambers were designed to be identical—with the exception that the sample chamber can accommodate the sample holder—the flow conditions within the chambers can be considered to be the same and thus the particle concentrations in the gas leaving the control and sample chambers can be compared.

To preserve the lifespan of the particle analyzer instrument and to rid the system of aerosol particles between individual tests, a jet of HEPA‐filtered air was used to purge the particle analyzer after each measurement and the sample chamber before swapping samples. See Figure S1 in the Supporting Information to see a schematic of the air flush system on the PFE apparatus.

To obtain a PFE measurement, three individual control readings were taken (i.e., from the control chamber; no material sample) followed by three individual sample readings (i.e., from the sample chamber containing a material sample), followed by three more control readings. The PFE was calculated from the average of sample readings, and the average of the control readings on either side of the sample readings. To account for the time that the system needs to reach equilibrium, we discarded the first (of three) control and sample measurements and reported PFE values which were composed of two sample and four control concentration values

(3) $$\begin{array}{c} \text{PFE}\;\left(\%\right)\;=\left[1-\frac{\text{average}\;\text{of}\;\text{two}\;\text{sample}\;\text{concentration}\;\text{readings}}{\text{average}\;\text{of}\;\text{four}\;\text{control}\;\text{concentration}\;\text{readings}}\right]\;\times 100\% \end{array}$$

This calculation represents a single “replicate” PFE measurement on a single material sample and is equivalent to the PFE calculation from ASTM F2299 (Equation (2)).

To compute the error associated with a single PFE replicate (σ_R_) the uncertainty in the individual particle concentration measurements was propagated forward^[^ ^23^ ^]^ according to Equation (4), where σ_c_ and σ_s_ are the standard deviations about the averages of the 0.10–0.15 µm control and sample particle concentration measurements, ${\bar{C}}_{c}$ and ${\bar{C}}_{s}$ respectively, and *p* is the aerosol penetration (see Equation (1))

(4) $$\begin{array}{c} \frac{\sigma_{R}}{p}\;\;=\;\;{\left[{\left(\frac{\sigma_{c}}{{\bar{C}}_{c}}\right)}^{2}+{\left(\frac{\sigma_{s}}{{\bar{C}}_{s}}\right)}^{2}\right]}^{0.5}\;\; \end{array}$$

PFE ± σ_R_ gives the variability (i.e., range) in measured PFEs for a single replicate.

Additional replicates were performed by replacing the material in the sample chamber with a new one and repeating the process; here, results were reported from three (triplicate) replicates. To propagate the variability within samples measured in triplicate, the propagated error (σ_prop_) value over multiple replicates was calculated,^[^ ^23^ ^]^ where σ_R,i_ is the error associated with the *i*th PFE replicate

(5) $$\begin{array}{c} \sigma_{\text{prop}}\;\;=\;\;{\left[\underset{i\;\;=\;\;1}{\sum }{\left(\sigma_{R,i}\right)}^{2}\right]}^{0.5}\;\; \end{array}$$

The total error associated with averaging the PFE measurements was reported from multiple replicates of the same material (σ_total_) as the maximum of either the propagated error (σ_prop_) or the simple standard deviation of the individual PFE replicate measurements (σ_sd_) about their average:

(6) $$\begin{array}{c} \sigma_{\text{total}}\;\;=\;\;\max\left\{\sigma_{\text{prop}},\sigma_{\text{sd}}\right\}\; \end{array}$$

The PFE averaged over multiple replicates is thus reported as (average PFE) ± σ_total_.

## 3 Results and Discussions

### 3.1 Measuring Filtration Efficiency of Mask Materials

**Figure 2:**
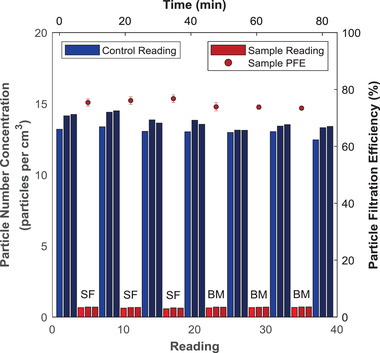
Raw particle number concentrations (bars) and calculated PFE values (points) for two potential mask materials. This time‐series plot shows the actual sequence of measurements taken in order to calculate the PFEs of the materials. Bars associated with sample measurements are labelled according to their materials of construction: SF = cotton/polyester sweater fabric and BM = batting material blend (87.5%/12.5% cotton/polypropylene). Error bars on the PFE points represent the error σ_R_ associated with a single replicate measurement.

The materials outlined in §2.1 were challenged with a latex aerosol using the PFE apparatus described in §2.3 to determine their suitability as mask materials. **Figure** **2** illustrates the readings obtained from the particle concentration analyzer while measuring the PFE value of the sweater fabric and batting material. Triplicate sample analysis is performed for a total of two materials and six replicates. It is evident that the first reading in a set of three readings (shaded with a lighter color) usually varies from the other two readings. For example, the first reading is more than 5% different than the average of the other two readings in 12–15 triplets in Figure 2. This observation is associated with the equilibration of aerosol‐laden air flowing through the particle analyzer. As discussed in the materials and methods, PFE was thus calculated using the equilibrated readings (second and third control and sample readings) shaded darker blue and red respectively, in Figure 2. Overall, the averaged PFE values over the three replicates are 78% ± 2% for the sweater fabric and 74% ± 2% for the batting material.

### 3.2 Screening Candidate Mask Materials

To benchmark the system's performance, ASTM 2100‐certified medical masks (L2, L3) and a NIOSH respirator (N95) were also evaluated. The ASTM F2100 standard benchmarks the sub‐micrometer particle filtration efficiency of 0.1 µm particles for Levels 2 and 3 masks at ≥ 95% and ≥ 98%, respectively.^[^ ^5^ ^]^ Similarly, NIOSH‐certified N95 respirators must show filtration efficiencies of ≥95% for aerosolized NaCl nanoparticles with a mean diameter of 0.075 µm.^[^ ^24^ ^]^ Rengasamy et al. reported that similar N95 masks demonstrated PFE values of 99.84%–99.98%,^[^ ^25^ ^]^ Zangmeister et al. reported that their N95s produced minimum filtration efficiencies greater than 99.9%,^[^ ^14^ ^]^ while Joo et al. reported that 3M N95 masks produced PFEs (300 nm particles) of 99%,^[^ ^16^ ^]^ and Hao et al. reported N95 PFEs (300 nm particles) of 94.4%.^[^ ^17^ ^]^ For the two N95 masks challenged with 0.1 µm latex spheres by Shakya et al., PFEs fell roughly in the middle of the range of 80% to 100%.^[^ ^19^ ^]^ When Lu et al. evaluated N95 masks with the same size of latex sphere, an average PFE of 93.8% was reported.^[^ ^20^ ^]^ The results obtained using the apparatus described in this work indicate that the N95 masks performed at a PFE of 99.2 ± 0.1%, which are in good agreement with most previous studies.

Surgical masks were also tested in several studies. Hao et al.'s surgical mask demonstrated PFEs (300 nm particles) of 73.4%,^[^ ^17^ ^]^ while three surgical masks tested by Drewnick et al. produced PFEs ranging from approximately 60% to almost 100%.^[^ ^15^ ^]^ (The latter mask is likely a certified model.) However, Zangmeister et al. reported that the two surgical masks that were tested demonstrated minimum filtration efficiencies of just over 30%,^[^ ^14^ ^]^ far less than the surgical masks tested in this study. Similarly, the “blue surgical mask” tested by Joo et al. demonstrated a PFE (300 nm particles) of only 22%.^[^ ^16^ ^]^ Shakya et al., reported that the PFE of a surgical mask (using 0.1 µm latex particles) fell roughly in the range of 60–80%.^[^ ^19^ ^]^ Using the same size latex spheres, Lu et al.'s surgical mask produced average PFE results of 80.2%.^[^ ^20^ ^]^ In comparison, our L2 masks performed at a PFE of 94.2 ± 0.6%, and the L3 masks performed at a PFE of 94.9 ± 0.3%. While these PFEs exceed many of those in the literature, we must note that there is little reference to the certification level in the previous studies, whereas our surgical masks were certified at Level 2 and Level 3.

**Figure 3:**
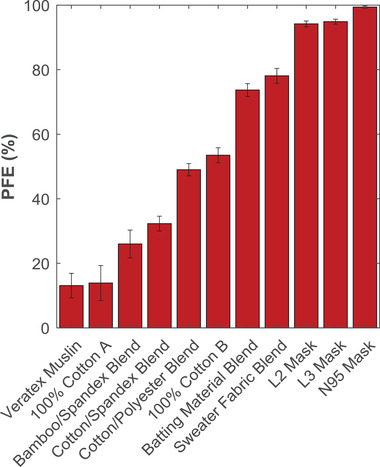
PFE data for eight fabric samples along with three certified medical masks. Each bar represents samples measured in triplicate, where the error bars represent the total error associated with measuring multiple replicates of a material, σ_total_.

In the same way that the candidate mask materials in Figure 2 were analyzed, six other materials were tested in triplicate to determine their suitability for use in face masks and to demonstrate the operation of the PFE apparatus. PFE values for all individual materials tested were plotted in **Figure** **3** and compared to PFE values obtained from evaluating certified L3 and N95 face mask materials. A wide range of PFE performance is evident from the eleven masks/candidate mask materials selected for this work, ranging from ≈10% to nearly 100%, coherent with other studies,^[^ ^8^, ^9^, ^15^ ^]^ and without clear relationships between industry descriptors and PFE. For example, materials with similar compositions (two 100% cotton samples and the cotton muslin), demonstrate vastly different PFE values (14%, 54%, and 13%, respectively). But critically, low‐performing (e.g., 100% Cotton A; PFE ≈ 14%), mid‐performing (e.g., 70%/30% cotton/polyester blend; PFE ≈ 49%), and high‐performing (e.g., sweater fabric; PFE ≈ 78%) fabrics can be clearly identified using the PFE apparatus described in this work. In particular, the sweater fabric and batting material both performed very well, with PFEs ranging from 70% to 80%. Such high‐performing materials can be identified and analyzed in greater detail to understand the properties that contribute to their high filtration performance. We do caution, though, that because of the wide variation in PFE results for materials with similar industry descriptors (i.e., cotton materials), these data do not suggest which materials are broadly useful for cloth masks, nor do we intend to infer property–performance relationships for classes of materials. We present concrete examples to demonstrate calibration, to show that measurements cover the whole range of possible filtration efficiencies, and to give a sense of the magnitude of measurement error.

We obtained these PFE results to demonstrate our apparatus’ wide range of measurement ability, low error, and to compare with data obtained in the literature. For example, Zangmeister et al. reported PFE results for several woven cotton fabrics (e.g., “Cotton 1–11”) (89 to 812 threads per inch). In nine of the eleven materials, the “minimum filtration efficiencies” ranged from 0% to 25%; the other two materials demonstrated PFEs of approximately 32%.^[^ ^14^ ^]^ Similarly, Konda et al. report PFE data for two cotton weaves (80 and 600 TPI; 153 and 152 g m^–2^) with PFEs ranging from 7% to 36% and 65% to 85%, respectively. Of the single‐layer cotton fabrics (i.e., cotton woven, cotton jersey, molleton; 139–265 TPI; 80–140 g m^–2^) in the study by Drewnick et al*.*, material PFEs ranged from ≈10% to 30%. In the study by Joo et al., their 100% cotton material gave a PFE of 8% (300 nm particles). Using polystyrene latex particles between 0.3 and 0.5 µm, Bagheri et al. measured the PFE of six cotton materials (120–200 TPI; 136–237 g m^–2^) which ranged from 0% to 20%.^[^ ^18^ ^]^ Thus, the majority of these materials agree well with our PFE results for the three cotton weaves (i.e., Veratex Muslin CT, Fabric Store Cottons A and B) which had average filtration efficiencies of 13%, 14%, and 54%. These results indicate wide variability between cotton materials and suggest that material properties which lead to high PFEs (i.e., Konda et al.'s 600 TPI cotton; our Cotton B) are poorly understood.

When making these comparisons, we do acknowledge that it is very difficult to find materials tested in the literature that have identical properties (i.e., material composition, weave versus knit, TPI, weight, etc.) to those tested in this study, so a direct comparison cannot be made. Furthermore, differences in the apparatuses used by the authors and a lack of standardization make good comparisons difficult. Still, it is clear that property/performance relationships for common fabrics are not yet strongly understood. Further testing of materials to determine these relationships will be enabled with a standardized, yet flexible and reliable apparatus like the one described in this work.

Despite the total statistical error within individual replicates (0–4%) and between samples analyzed in triplicate (0–5%), the apparatus presented in this work proves to be an effective tool for testing the PFE of various materials, from common fabrics to certifiable medical masks. Notably, of the eleven materials tested for Figure 3, the propagated error σ_prop_ exceeded the standard deviation between the PFE measurements of the individual samples, σ_sd_ in nine of the eleven materials; the two exceptions occurred at very high PFE values (i.e., L2 and L3 masks). While the results presented by Rengasamy et al. show less variability between replicate samples (i.e., <0.29% for five replicates),^[^ ^25^ ^]^ they studied materials with high known filtration performance designed for mask manufacture: the materials themselves may have been more uniform and testing also may have been more consistent in this region of the PFE range. Overall, the results obtained using our apparatus align favorably with PFE data obtained by other researchers and with the certification standards.

While PFE is an important measure of mask performance, at this point we must caution the reader that a thorough analysis of prospective mask materials must take other factors into consideration, namely the material breathability (i.e., via pressure drop or differential pressure test), which is prescribed in both ASTM F2100 and F3502. Acceptable breathability is crucial for wearer comfort and for preventing leakage around the edges of the face covering during respiration. As PFE and breathability can often be inversely related for many common materials, pressure drop measurements should be performed alongside PFE measurements for a more complete assessment of mask material performance.

### 3.3 Design of the PFE Apparatus and ASTM Standards

We suggest that guidance for building PFE devices along the lines of ASTM F2299 is crucial to continually improving standards, generating research data that is comparable across research labs, and enhancing the field of aerosol filtration. Sole reliance on the NIOSH (or F3502) standard, which specifies a single piece of equipment (the TSI 8130A), constrains researchers to purchasing a turn‐key device (e.g., the TSI system). The dependence on standardized systems such as the TSI 8130A is important for certification to current standards, but limits the development of face masks, respirators, and other aerosol filtration technologies which is antithetical to research progress. It is important to note that the NIOSH standard was developed as a method for testing respirators under the harsh conditions that one would expect when needing such a device but in contrast, surgical masks are tested via the ASTM F2100/F2299 method. Community‐use face masks are more like the surgical masks in their shape and style, and that they are not meant to have the superior filtration efficiency performance like that seen in N95s. If surgical masks are still evaluated under ASTM F2100/F2299, it follows that common fabrics should be analyzed using a method closer to the ASTM F2100/F2299. Furthermore, ASTM F2299 allows for additional flexibility in varying parameters such as air flow rate and face velocity in filtration efficiency studies which may make it a superior standard to approximate in research environments.

Our choice of using latex spheres (as per ASTM F2299 vs ASTM F3502 or NIOSH) as virus‐like particles stems from adherence to the ASTM F2299 standard, but also the observation that the latex more closely mimics the composition of actual viruses as the density of a single virus is more similar to the density of latex than to the density of a salt crystal. All other things equal, differences in particle density and charge of similarly‐sized NaCl or latex particles may affect the mechanism of capture. In addition, it should be noted that the “median count diameter” of the NaCl particles from the NIOSH standard are 0.075 µm while the latex particle size from ASTM F2299 is 0.1 µm. In terms of size, ASTM F2299 better approximates the size of a “naked” COVID‐19 virus (≈0.11 µm)^[^ ^14^ ^]^ and may better represent the most challenging particle size to filter (roughly 0.1–0.3 µm).^[^ ^26^, ^27^ ^]^

Further research is needed to align our knowledge of the composition of respiratory droplets with personal protective equipment standards.

Designing such a custom‐built system allows researchers to explore the impact of various parameters and evaluate the appropriate parameters for a specific application (e.g., approximating the size of aerosolized viruses that originate from the lungs) at a cost which is less than turn‐key commercial systems. We estimate the costs of building the equipment we describe at $40 000–$55 000 USD ($50 000–$70 000 CAD), excluding labor, which is likely significantly lower than the purchase price of a TSI 8130A.

However, the guidelines provided by ASTM F2299 are unclear and offer large ranges for parameters within which they are deemed acceptable. These large ranges promote wide variability between research labs, which prevents proper comparison of results from different research groups. Additionally, in our experience, industry typically tends to use parameters that facilitate certification. For example, ASTM recommends air flow rates between 1 and 1000 L min^–1^. In our experience, masks tend to exhibit better PFEs at lower flow rates, thus industry measurements favor the minimum flow rates recommended by ASTM, making the higher flow rates undesirable from the point of view of a mask manufacturer. These ranges of parameters are tightened in the more recent F3502 standard, but the requirement for the TSI apparatus is still onerous.

As such, we recommend that standards facilitate custom built designs as per ASTM F2299, while clearly identifying parameter values as per NIOSH and ASTM F3502. In this way, standards are established such that all research labs and testing facilities can compare their baseline results, while enabling the exploration of other parameters and their impact on the effectiveness of various materials and material configurations. The design of the system in this work attempts to match the requirements set out in the two aforementioned ASTM standards, while providing flexibility to investigate various filtration parameters. A summary comparing systems that are ASTM‐ and NIOSH‐compliant to the apparatus described in this work is presented in **Table** **2**. With exception to charge neutralization and air temperature/humidity control, the apparatus described in this work fits the requirements within the ASTM F2100 and F2299 documentation.

**Table 2:** Comparison of key specifications from the ASTM and NIOSH methodologies for mask testing. A summary of the corresponding parameters in the McMaster PFE apparatus is also included

Specification	ASTM F2100/F2299	NIOSH [ASTM 3502]^a)^	McMaster PFE
Recommended Apparatus	None	TSI Model 8130 or 8130A	Custom
Aerosol Particle Composition	Dry latex spheres	Dry NaCl particles	Dry polystyrene latex spheres
Aerosol Particle Properties	F2299: 0.1–5 µm spheres F2100: 0.1 µm particles	Count median diameter of 0.075 ± 0.020 µm; geometric standard deviation ≤ 1.86	0.125 ± 0.025 µm spheres
Aerosol Particle Concentration	<10^2^ particles cm^–3^; specifically 10–100 particles cm^–3^	<200 mg m^–3^	(<34 particles cm^–3^)^b)^
Aerosol Generation	Various atomizers	Built‐in (e.g., TSI Model 8118A)	Blaustein aerosol generator
Charge Neutralization	Recommended	Yes	No
Air Pretreatment	HEPA filtration, moisture, and oil removal	Not specified in standard^d)^	HEPA filtration; aerosol drying
Apparatus Air Temperature and Humidity	Temperature: not specified Humidity: 30–50 ± 5%	Temperature: 25 ± 5 °C Humidity: 30 ± 10%	Ambient temperature and ambient humidity (30–40%)^c)^
Air Flow Rate	1–1000 L min^–1^	1 filter: 85 ± 4 L min^–1^ 2 filters: 42.5 ± 2 L min^–1^ 3 filters: 28.3 ± 1 L min^–1^ [85 ± 4 L min^–1^]	1 CFM (28.3 L min^–1^)
Average Air Face Velocity at Mask	0.5–25 cm s^–1^	Not specified in standard^d)^; 17.3 cm s^–1^ for a 102 mm diameter flat, circular sample [flat sample: 10 ± 0.5 cm s^–1^]	11.3 cm s^–1^
Test Section/Sample Diameter	50–150 mm	Compatible with TSI filter tester	73 mm
Upstream Test Section Length	>10 duct diameters (i.e., 500–1500 mm)	Not specified in standard^d)^	>10 duct diameters (≈740 mm)
Material Preconditioning Parameters	Temp.: 21 ± 3 °C Humidity: 30–50 ± 5%	Temp.: 38 ± 2.5 °C Humidity: 85 ± 5% Duration: 25 ± 1 h	Ambient temperature and humidity^c)^
Sampling	Isokinetic via probe sampler	Not specified in standard^d)^	Completely isokinetic; entire stream sampled
Sample Analysis	Single‐particle light‐scattering counter	Upstream and downstream photometers	LASAIR III 110 Laser Particle Counter

^a)^ The ASTM F3502 parameter is included (in square brackets) where it differs from what is prescribed in the NIOSH standard;

^b)^ Below the maximum cumulative particle concentration measurable by the laser particle analyzer;

^c)^ “Ambient” temperature ranges 20–25 °C in the laboratory. Air in the apparatus contains a very small amount of additional humidity added when the water sheath surrounding the latex particles is evaporated;

^d)^ The specification is not explicitly described but satisfied by virtue of using the apparatus (TSI Model 8130 or 8130A) specified by the standard.

### 3.4 Efficiency and Flexibility of the PFE System

One of the advantages of designing a custom‐built system is the ability to investigate filtration parameters outside those dictated by the standards, while also designing an efficient system for rapid testing. The PFE apparatus described in this work is able to process samples efficiently. To obtain the data presented in Figure 2, six replicates were completed in 80 min. This corresponds to an average analysis time of 14 min per replicate and 42 min per material analyzed in triplicate. This demonstrates that the PFE system is a useful tool for quickly screening the filtration efficiency of materials. Rengasamy et al. indicate that the NIOSH standard involves a test duration of 90–100 min to complete an individual replicate due to the particle loading prescription in the NIOSH standard; however, they also appear to recognize the value in rapid testing of materials as they employed an abbreviated version of their test which required ≈15 minutes per respirator,^[^ ^25^ ^]^ similar to our average analysis time.

Furthermore, a custom‐built PFE system permits manipulation of system variables of interest, for example:

- The effect of particle composition on filtration performance. Current standards prescribe the use of different simple particle chemistries including latex,^[^ ^4^, ^5^, ^11^ ^]^ salt,^[^ ^6^, ^7^, ^12^, ^13^ ^]^ or oil,^[^ ^12^ ^]^ yet it is understood that actual respiratory droplets are complex mixtures containing varying amounts of water, salts, cholesterol, proteins, enzymes, surfactants, cell debris, and pathogens.^[^ ^28^, ^29^ ^]^ The effect of droplet composition on filtration performance is not well understood, but we suggest that future PFE experiments could use mixtures of solutes, with initial experiments measuring the impact of using salt and latex as a virus mimic. Using the apparatus described in this work, it would be trivial to aerosolize particles from solutions of varying chemistries to elucidate the effect of particle composition on aerosol penetration. The flexibility of the Blaustein‐type aerosol generator would allow for any of these types of particles to be aerosolized and subsequently transported to the sample. This facilitates the detailed study of how particle chemical composition affects PFE results. Notably, the apparatus described in this work is ideal for performing this sort of measurement as it can infuse both types of aerosolized particles without changing the system.
- The effect of aerosol particle size on filtration performance. It is well‐known that the mechanism of particle removal by fibrous materials (diffusion, diffusion–interception, interception, impaction, sedimentation), as found in face masks, is related to the particle size of the aerosol.^[^ ^15^ ^]^ Multiple studies have characterized the PFE of aerosol particles of different sizes and generally conclude that larger particles are removed in higher proportions, presumably because of greater interception and impaction of particles on fibers within face masks.^[^ ^8^, ^14^, ^15^, ^16^, ^17^ ^]^ The particle analyzer used in our study has the ability to measure particles from 0.1 to 5 µm in size, sorted into several user‐defined bins. This allows filtration measurements and study of various particle sizes beyond those defined by standards.
- Particle loading of the mask. To investigate the effect of the cumulative number of particles that reach the mask on filtration performance, the syringe pump infusion rate (or the latex suspension concentration) can be varied to change the number of particles reaching the sample. The upper limit is governed by the maximum number of particles (≈950 000 per cubic foot across all bin sizes) which is reliably read by the particle analyzer. Sampling time may also be easily varied.
- The effect of “wet” versus “dry” aerosols. If very large aerosol particles are generated, switching the aerosol heater on/off may allow the particles to dry or remain surrounded by water. This provides a useful comparison of the filtration of aerosols from a fresh, “wet” sneeze versus aerosols that have dried. Overall, the drying dynamics of respiratory particles is not well understood, especially given the complex makeup of these particles.^[^ ^28^ ^]^ Further research is needed to understand how particle desiccation affects filtration performance.
- Face velocities. The flow rate of air to the mask (see Table 2), 28.3 L min^–1^ is dictated by the flow rate required by the particle analyzer. Changes to the apparatus (i.e., the addition of additional purge valves, isokinetic sampling tubes, etc.) could produce other flow rates. However, the sample holder within the system is entirely removable and replaceable (see Figure S2 in the Supporting Information). The air face velocity can therefore be increased by inserting a sample holder with a smaller surface area of material exposed to the air stream, that is, a sample holder with a smaller aperture. Should stretching of the sample be deemed a concern—for example at high face velocities—a mesh screen could easily be laid under samples, as outlined in ASTM F3502.^[^ ^7^ ^]^

To further refine our PFE apparatus, a charge neutralizer will be added downstream of the aerosol generator in the very near future. This creates a “worst‐case scenario,” eliminating particle removal due to electrostatic attraction or repulsion between charged particles and the mask material. There is still debate on how much (if any) charge is present on aerosolized viruses or respiratory particles, or how this surface charge might dynamically evolve over time, for example, with desiccation.^[^ ^30^, ^31^, ^32^ ^]^ The US Food and Drug Administration allows certification of masks according to ASTM 2100/F2299 standards using neutralized (as recommended in ASTM F2299) or un‐neutralized particles.^[^ ^33^ ^]^ The NIOSH standard (and ASTM F3502 by extension), in contrast, requires a neutralized NaCl aerosol.^[^ ^6^ ^]^ We plan to compare neutralized with un‐neutralized PFEs to ascertain the importance of charge neutralization. Furthermore, temperature and humidity control/monitoring of the air in the apparatus will be performed using inline sensors (as per the ASTM standards) along with sample preconditioning to investigate the impact of temperature and humidity on PFE.

## 4 Conclusions

With the ongoing COVID‐19 pandemic, there is an urgent need to evaluate candidate mask materials for filtration performance. The PFE tests from ASTM International and the NaCl aerosol challenge test by NIOSH are two of the most common North American methodologies by which mask PFE can be measured reliably. Prebuilt equipment to perform these tests presents high up‐front costs, while external laboratories charge high prices to evaluate a small number of samples. We have designed and built an apparatus based on the ASTM F2299 standard. Currently, the results proceeding from this standard are underrepresented in the literature. The system was constructed from relatively common and nonspecialized equipment; the details for building the apparatus are presented herein. Using aerosolized 0.1 µm polystyrene latex particles, the PFE values of eleven fabrics and masks were reliably measured. The results ranged from ≈10% (muslin; 100% cotton) to nearly 100% (N95 mask), which demonstrates the wide range of filtration efficiencies measurable by the PFE apparatus. With each sample replicate requiring ≈14 min to test, our methodology is efficient when screening large libraries of samples. Future work will employ the apparatus to evaluate a range of common fabrics and commercially available face masks for their filtration performance. Overall, we recommend that other researchers utilize this design to iteratively evaluate their novel PPE designs and materials. We recognize that the PFE of materials for non‐medical face masks, measured by the standards of ASTM and NIOSH, will be low. This will create a need for public education around the principle of testing and design towards a worst‐case, viral‐size particle, and the appropriate interpretation of results. However, we believe that consistency between standards and the advantage of using a familiar standard is more important than choosing a particle size that is filtered more efficiently. We therefore support the creation of filtration and breathability standards for non‐medical face masks that build off well‐understood standards for PPE, such as the NIOSH and ASTM standards described here in detail.

## Conflict of Interest

The authors declare no conflict of interest.

## Supporting information

Supporting Information

## Data Availability

Research data are not shared.
