# Supplementary material for: Navigating Performance Standards for Face Mask Materials: A Custom‐Built Apparatus for Measuring Particle Filtration Efficiency
Source: Glob Chall. 2021 Aug 5;5(11):2100052. doi: 10.1002/gch2.202100052 (PMC8420507; doi:10.1002/gch2.202100052)
Supplement: Supplementary file 1 — Supporting Information [file GCH2-5-2100052-s001.pdf]

© 2021 Wiley-VCH GmbH

Global Challenges
Open Access

Supporting Information

for *Global Challenges,* DOI: 10.1002/gch2.202100052

Navigating Performance Standards for Face Mask Materials: A Custom-Built Apparatus for Measuring Particle Filtration Efficiency

*Ryan J. LaRue, Patrick Morkus, Scott Laengert, Sarah Rassenberg, Mohamad Amin Halali, John W. Colenbrander, Catherine M. Clase, David R. Latulippe, and Charles-Françoisde Lannoy**

SUPPLEMENTARY INFORMATION

S1. Comparison of Prevailing Standards—Full Text

There are several established standards associated with evaluating medical mask particle PFE; adoption varies by jurisdiction. The methodologies within vary substantially, including parameters such as the particle chemical composition (*e.g.* latex, [4], [5] salt[6], [7]), size (*e.g.* 0.1 µm,[4] 0.1–5 µm,[5] 0.075 ± 0.020 µm[6], [7]), and concentration (*e.g.* 10–100 particles cm^{-3} and < 100 particles cm^{-3},[4] < 200 mg NaCl m^{-3} [6]), as well as test conditions such as the air flow rate (*e.g.* 1–1000 L min^{-1},[4] 85 ± 4 L min^{-1} [6], [7]), face velocity at the sample (*e.g.* 0–25 cm s^{-1},[4] unspecified[6], 10 cm s^{-1} [7]), and temperature/humidity.[4]–[7] Performance benchmarks also vary. Navigating these standards can prove to be confusing for researchers; the following section serves to clarify and compare the prescriptions of several major standards.

S1.1. NIOSH Procedure TEB-APR-STP-0059: Determination of Particulate Filter Efficiency for N95 Series Filters Against Solid Particulates for Non-Powered, Air-Purifying Respirators Standard Testing Procedure (STP)

This procedure (Revision 3.2, December 13th, 2019) is designed for the certification of N95 candidate respirators, but not non-medical face masks.[6] §3.1.1 specifies the use of a TSI Automated Filter Tester Model 8130 or 8130A system (or equivalent) built by TSI Incorporated. This dramatically reduces the number of design variables as the required system is prebuilt. In the following, details not outlined in the NIOSH standard are substituted from specifications of the TSI instrument provided by the manufacturer.

- **The aerosol particles.** The aerosol is produced from a 2% solution of sodium chloride in distilled water (§3.1.5). The particle size distribution of this aerosol is to have a “count median diameter of 0.075 ± 0.020 micrometer and a geometric standard deviation not exceeding 1.86” (§5.1). The standard does not explicitly state whether the aerosol “particle” should be a liquid particle, or if it should be composed of a solid nanoparticle of crystallized NaCl, but the standard’s title infers that the intention is solid particulates. As found in manufacturer documentation, an air heater is included in the TSI instrument to dry the produced aerosol.

- **Aerosol generation.** No aerosol generator is specified in the standard because the device is built into the TSI system. From the product literature, a Model 8118A aerosol generator recommended for the Model 8130A test apparatus typically produces a concentration of aerosol particles in the range of 12–25 mg m^{-3}, under the upper limit of particle concentration (200 mg m^{-3}) set in §5.1. The final particle to be filtered by the mask material is smaller by NIOSH standards (0.075 µm) than that stipulated by ASTM (0.1 µm).
- **Aerosol neutralization.** The aerosol is to be "neutralized to the Boltzmann equilibrium state" (§5.1), which is achieved via a high voltage aerosol neutralizer in a typical TSI 8130 instrument.
- **Air supply.** No explicit prescriptions are made, but TSI 8130A product literature specifies that feed air at 80 psi (550 kPa) is required with a flow rate of 198 standard L min^{-1}.
- **Sample testing apparatus.** No specific holder is prescribed onto which the "filters will be mounted and sealed" (§5.3), but a "test fixture [which] is supplied by the applicant" is allowed to accommodate varying sample geometries (§4.2.4). The exact testing procedure that is to be performed to evaluate samples is stated in §5.4.
- **Temperature and Humidity.** The aerosol sent to the sample should have a temperature and humidity of 25 ± 5°C and 30 ± 10%, respectively, as measured in the system exhaust (§5.1).
- **Aerosol velocity/flow rate.** The standard considers full respirators/masks, including respirators with multiple cartridge filters. Specific flow rates are used according to the construction of the mask: 85 ± 4 L min^{-1} for a single filter, 42.5 ± 2 L min^{-1} for two filters, and 28.3 ± 1 L min^{-1} for three filters. Face velocity is not specified as it is determined by the geometry of the sample holder. For a typical full respirator (single filter; area ≈ 150 cm^2), this corresponds to a value of roughly 10 cm s^{-1}.
- **Aerosol sampling.** No explicit prescriptions are made; sampling is handled by the TSI instrument.
- **Sample conditioning and analysis.** Samples should be "pre-conditioned at 85 ± 5% relative humidity and 38 ± 2.5^{o}C for 25 ± 1 hours", with testing occurring within 10 hours (§5.2). A set of light-scattering photometers—upstream and downstream of the

sample—are used to measure the concentration of aerosol particles before and after the sample (§5.1.4). The penetration (*i.e.* downstream concentration divided by the upstream concentration) is then reported by the instrument and filtration efficiency can be obtained via Equation S1:

$$\text{efficiency} = [1 - \text{penetration}] \times 100\% \qquad \textbf{(S1)}$$

S1.2. ASTM International Standards

As outlined below, three ASTM standards describe the design of systems to measure the PFE in materials for use in masks:

S1.2.1. ASTM F2100-19e1: Standard Specification for Performance of Materials Used in Medical Face Masks

The F2100 standard (approved in 2001, latest update 2019), is used to test the materials for use in medical facemasks, certified according to "levels" 1–3 (L1, L2, or L3).[5] ASTM F2100 provides an overview of the performance of a mask material using five tests to evaluate different performance characteristics, one of which is the "sub-micron particulate filtration efficiency test". ASTM F2100 references ASTM F2299 for the methodology by which to conduct a PFE measurement. ASTM F2100 assigns masks a "level" based on how they perform in the five included tests. Level 1 masks exhibit a sub-micron PFE of ≥ 95 percent, and Levels 2 and 3 masks exhibit a sub-micron PFE of ≥ 98 percent. Notably, ASTM F2100 defines that the sub-micron PFE is measured using 0.1 micron particles (§6.1), with no composition or shape specified.

S1.2.2. ASTM F2299 / F2299M-03(2017): Standard Test Method for Determining the Initial Efficiency of Materials Used in Medical Face Masks to Penetration by Particulates Using Latex Spheres

The F2299 standard, (approved in 2003, latest updated 2017), seeks to "establish procedures for measuring the initial particle filtration efficiency of materials used in medical face masks using monodispersed aerosols […] in the size range 0.1 μm to 5.0 μm".[4] Neither ASTM 2100 nor the subset standards, such as ASTM F2299, intend to be "comprehensive characterizations" (§5.2.2),

such that they do not consider design or fit of the face masks, or the analysis of non-medical face mask performance. A summary of the main points in the standard can be found below:[4]

- **The aerosol particles.** Aerosols are defined to be "solid particles having a diameter of 0.1 to 5 μm suspended or dispersed in an airflow at concentrations of less than 10^2 particles/cm^3" (§3.1.1.1). The distribution should be monodisperse latex spheres (§3.1.3.1; §4.1), but no further composition details such particle chemical composition and surface chemistry/charge are specified. Note that while ASTM F2299 details how PFE can be performed using particles between 0.1–5 μm, ASTM F2100 specifies that medical facemasks must be tested with 0.1 μm particles.
- **Aerosol generation.** The aerosol generator that is chosen "must be capable of a latex sphere count concentrations output of 10^7 to 10^8 particles/m^3" (10–100 particles/cm^3; §7.2.1). The standard suggests "atomizing suspended uniform latex spheres from dilute water suspensions" (§7.2.2) by diluting "10 %-by-volume solids […] at 1000:1 or greater dilution ratios in deionized, filtered distilled water". Footnote 1 recommends dilution on the order of 1000:1 to 10,000:1. From a 10% initial suspension, this corresponds to 0.1–0.01 ppm latex feeds. The standard recommends collision-type atomizers specifically, a Collison-type atomizer as well as Blaustein-type atomizers for testing (§7.2.2).
- **Aerosol neutralization.** ASTM F2299 calls for the neutralization of surface charge on the aerosol prior to injection into the test system with a "typical ionizing flux of 10^3 mCi/m^3/s" (§7.3) such as that provided by a ^{85}Kr, ^{210}Po, or corona discharge source.
- **Air supply.** The standard reinforces the need for a clean air supply to limit background aerosol particles in the test. Silica gel driers for moisture removal, molecular sieves for oil removal, and a high-efficiency filter (*e.g.* a HEPA filter; §7.6.2) for removal of latent aerosols are recommended (§7.1), especially for the feed to the aerosol generator. The standard requests temperature and humidity monitoring.
- **Sample testing apparatus.** The "material specimen holder and test section shall be a continuous straight-walled vessel" (§7.5.1) with a cross-sectional diameter of 50 to 150 mm (2 to 6 in). The aerosol-in-air stream is added to makeup air to achieve the final concentration and flow rate (§7.4) . The test section is to extend at least 500 to 1,500 mm upstream of the sample (§7.5.1) and the apparatus should be "mounted in the vertical

orientation to minimize aerosol sedimentation losses" (§7.1.1). There should be negligible pressure drop across the sample holder, as measured via static pressure taps.

- **Temperature and Humidity.** Testing of samples "should be performed in a relative humidity range of 30 to 50 %, held within ± 5 % during a given test" (§7.4), but there is no temperature prescription.
- **Aerosol velocity/flow rate.** The apparatus should provide face velocities ranging from approximately 1 to 50 ft min^{-1} (0.5 to 25 cm s^{-1}) given volumetric air flow rates ranging from approximately 0.035 to 35 cubic feet per minute (1 L min^{-1} to 1 m^3 min^{-1}), which constrains the design of the system with respect to the test section diameter. Note the wide range of acceptable face velocities and air flow rates through the sample, which have 50-fold and 1000-fold ranges, respectively.
- **Aerosol sampling.** It is prescribed that "geometrically and kinematically identical centerline probes [are] to extract representative aerosols from the inlet and outlet sides of the material specimen test section" (§7.8). These probes are to "have a radius of curvature (R) of 12 cm or R/D [radius/diameter] > 20:1 and present a cross-sectional area of less than 10 % of the cross-sectional area of the test system ducting." The probe upstream of the sample is to be located a minimum of 8 duct diameters downstream of the aerosol sample injection, and two duct diameters upstream of the sample. Downstream, the probe is to be located three duct diameters away from the sample. The lengths of "horizontal sample line" and "total sample transport line" is limited to 1 and 2 meters, respectively. Within, the sample flow rate should not exceed 10% of the "total test system flow rate" (§7.8.3), with sampling occurring in the laminar flow region. The Reynolds Number (*Re*) is to be less than 1,000 within the sampling lines (§7.8.1). The dimensionless value of *Re* is defined as follows, where ρ is the air density (kg m^{-3}), v is the average velocity of the air (m s^{-1}), d is the circular duct diameter (m), and μ is the air viscosity (Pa s):

$$Re = \frac{\rho v d}{\mu} \qquad \textbf{(S2)}$$

  Where *Re* cannot be less than 1,000, other provisions are detailed.
- **Sample conditioning and analysis.** Samples should be preconditioned at "30–50 ± 5 % relative humidity and a temperature of 21 ± 3 ^{o}C [50 ± 5 ^{o}F]" (§9.5). A material specimen mounted in the sample chamber is then subjected to an equilibrated stream of aerosol in

air. To analyze the isokinetically-sampled streams drawn from the probes, a calibrated "automatic, single particle light-scattering counter" (§7.9.1) is to be used for aerosol concentration measurements before and after the material specimen. The efficiency by which the material rejects the aerosol particles is calculated as follows (§10.4.6):

$$\text{efficiency} = \left[1 - \frac{\text{average downstream particle concentration}}{\text{average upstream particle concentration}}\right] \times 100\% \qquad \textbf{(S3)}$$

S1.2.3. ASTM F3502-21: Standard Specification for Barrier Face Coverings

The new F3502 standard, last updated February 15th 2021, was created in response to the COVID-19 pandemic, and the need for standardized masks for the general public.[7] Unlike previous standards, it seeks to issue guidelines for generic face coverings (not just "medical masks") with specifications including "design criteria, […] labelling, and user information" (§1.2.5), in addition to the traditional performance evaluation and test methods. Design features, sub-micron particle filtration efficiency, breathability, leakage and fit, and considerations if the mask is reusable/washable are all prescribed; as such, this standard is much more comprehensive than previous iterations. For filtration performance benchmarks under ASTM F3502, "Level 1" certified masks will have sub-micron PFEs measured at 20% or more, which increases to 50% or more for "Level 2" certified masks.

With regards to this filtration efficiency testing, ASTM notably supplants their own PFE testing procedure in F2299 in favor of NIOSH's TEB-APR-STP-0059 test procedure, and therefore the TSI 8130/8130A system. That is, the PFE test outlined in F3502 "poly-disperse sodium chloride (NaCl) aerosols with a count median diameter of 75 ± 20 nm electrical mobile diameter and a geometric standard deviation of ≤1.86 to give a mass median aerodynamic diameter of 0.3 μm" (§4.1.1), as is the case in the NIOSH standard. However, F3502 makes the following notable additions or modifications to the NIOSH procedure with respect to the testing apparatus and conditions:[7]

- **Sample testing apparatus.** It is specified that the sample holder should contain a mesh screen (70% or more open area), a wire frame, or another such device to support samples that lay flat against the filter holder so to "prevent [the] collapse of the product into the

equipment, which can potentially affect test results" (§8.1.2.1). The sample must be mounted in a way that prevents leakage, where seals can include "hot melt glue, beeswax, rope caulk […], or another appropriate sealant" (§8.1.2.4). Then, a small chamber is to be created around the upstream side of the sample, which is then sealed to the rest of the apparatus with gasket material (§8.1.2.5).

- **Aerosol velocity/flow rate.** It is prescribed that samples "shall be tested at a flow rate of 85 ± 4 [L min^{-1} of air]" (§8.1.3.5), but if the sample "lays flat in the filter holder, adjust the flow rate to achieve a face velocity of 10 ± 0.5 cm/s."

As is the case with the NIOSH standard, particle concentrations are to be "determined by using a forward-light-scattering photometer or equivalent" (§8.1.3.3), and PFE is to be calculated according to Equation S1, as before.

S1.3. Other Standards

Other organizations have published their own versions of mask testing standards, some of which have been created to meet the increased facemask needs during the COVID-19 pandemic. In particular, standards have been produced for non-medical face masks by the American Association of Textile Chemists and Colorists (AATCC),[11] the Association Française de Normalisation (AFNOR) from France[12] and the Bureau de normalisation (BNQ) from Quebec[13], all of which parallel—to some degree—either the NIOSH or ASTM standards.

AATCC has issued guidelines in their "Guidance and Considerations for General Purpose Textile Face Coverings: Adult" document which outlines the test procedures for the face masks.[11] Their standards piggyback off the ASTM F2299 standard while specifying that the filtration efficiency is to be measured using 3 µm latex spheres (with an option for smaller sizes) carried to the material at a specified air face velocity of 10.4 cm s^{-1}. Mask materials should not have a filtration efficiency lower than 70%—much less than the medical mask certification "levels" in ASTM F2100, but higher than the "levels" in ASTM F3502.

Similarly, AFNOR developed a standard in mid-2020 to address the need for standards on non-medical face masks. This standard tests face masks with either an aerosolized NaCl solution or paraffin oil and allows a range of sizes up to 3.0 µm. They require face masks to be able to filter at least 70% of these aerosolized particles.[12]

In contrast, the BNQ standard more closely mimics the NIOSH standard. It requires 20–4,000 nm dried and neutralized NaCl particles which are diluted with compressed air and fed to the sample at a volumetric flow rate of 20.7 L min^{-1}, corresponding to a superficial air velocity of 27.45 cm s^{-1} at the material sample, having a diameter of 4 cm. The standard calls for PFE ≥ 80% for 20–800 nm particles, and ≥ 95% for 3,000 nm particles.[13] This generally exceeds the PFE requirements in ASTM 3502.

S2. Design of PFE Apparatus

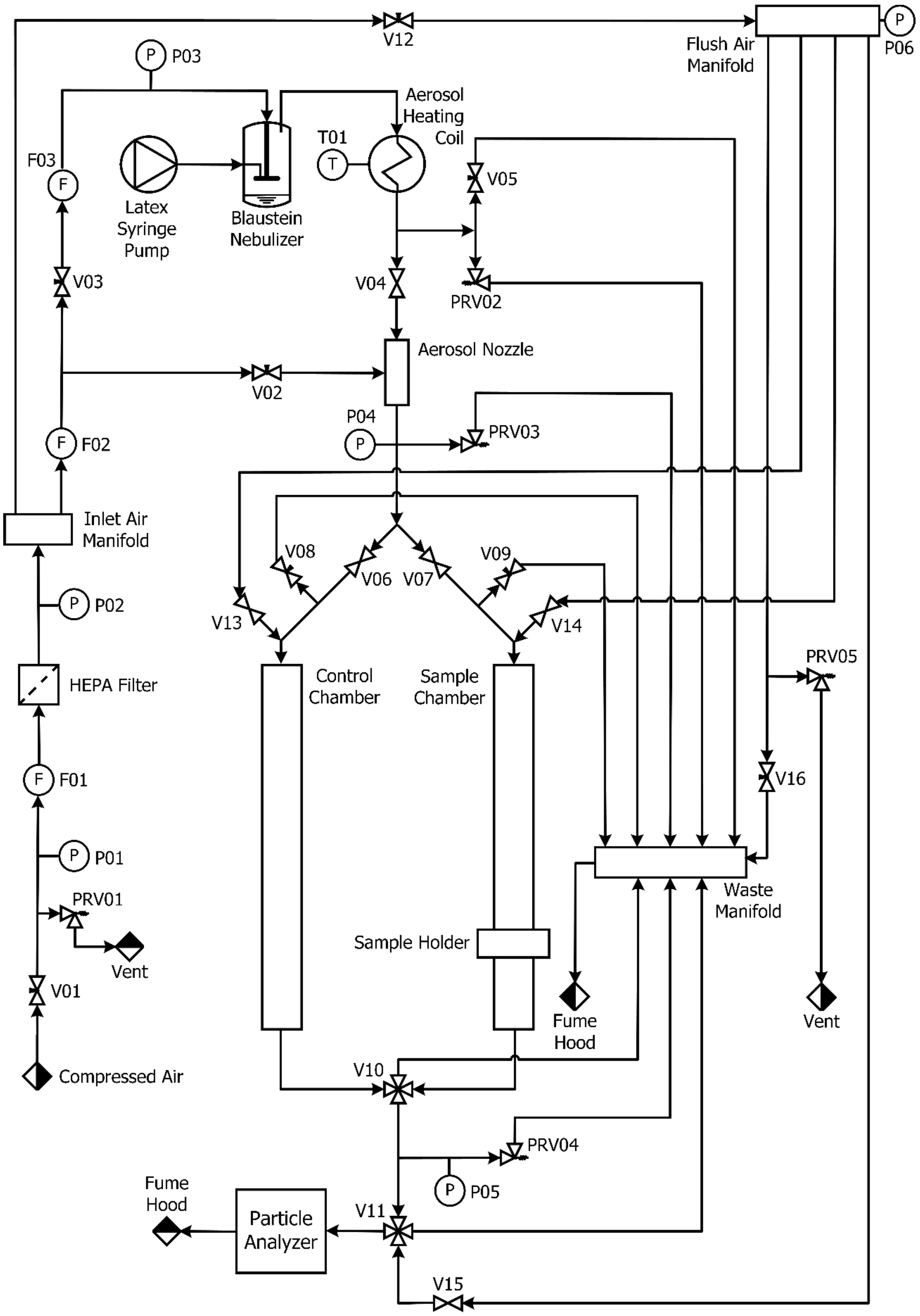

Figure S1: Piping and instrumentation diagram of the McMaster PFE system. Valve and gauge annotations are summarized in Table S1.

Table S1: Tables of valves and instrumentation for the McMaster PFE system. The valve and instrumentation nomenclature refers to the P&ID annotations in Figure S1.

Manual Valves	
V01	Inlet Air Valve
V02	Makeup Air Valve
V03	Nebulizer Air Valve
V04	Nebulizer Isolation Valve
V05	Aerosol Purge Valve
V06	Control Chamber Valve
V07	Sample Chamber Valve
V08	Control Dump Valve
V09	Sample Dump Valve
V10	Selector (4-Way) Valve
V11	Analyzer (4-Way) Valve
V12	Flush Air Valve
V13	Control Flush Valve
V14	Sample Flush Valve
V15	Analyzer Flush Valve
V16	Sweep Air Valve

Temperature Gauges	
T01	Aerosol Heating Coil Temperature

Pressure Relief Valves	
PRV01	Inlet Air Pressure Relief Valve
PRV02	Nebulizer Pressure Relief Valve
PRV03	Aerosol Nozzle Pressure Relief Valve
PRV04	Analyzer Pressure Relief Valve
PRV05	Flush Air Pressure Relief Valve

Pressure Gauges	
P01	Inlet Air Pressure
P02	Feed Air Pressure
P03	Nebulizer Pressure
P04	Aerosol Nozzle Pressure
P05	Analyzer Pressure
P06	Flush Air Manifold Pressure

Flow Meters	
F01	Inlet Air Rotameter
F02	Feed Air Mass Flow Meter
F03	Nebulizer Air Rotameter

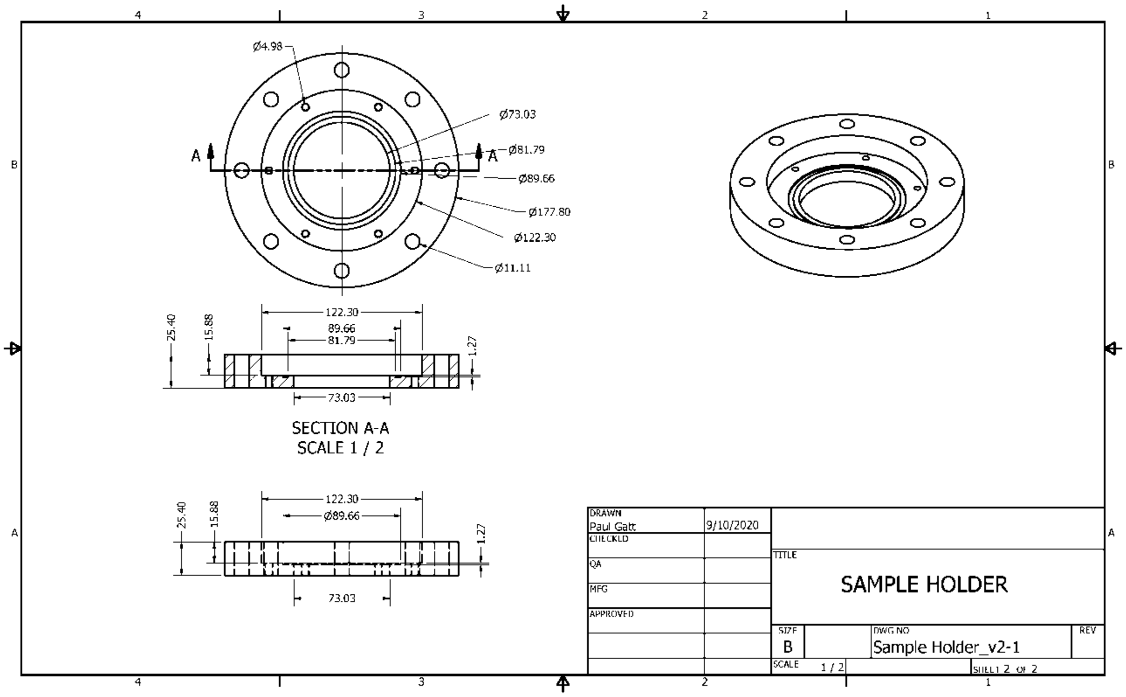

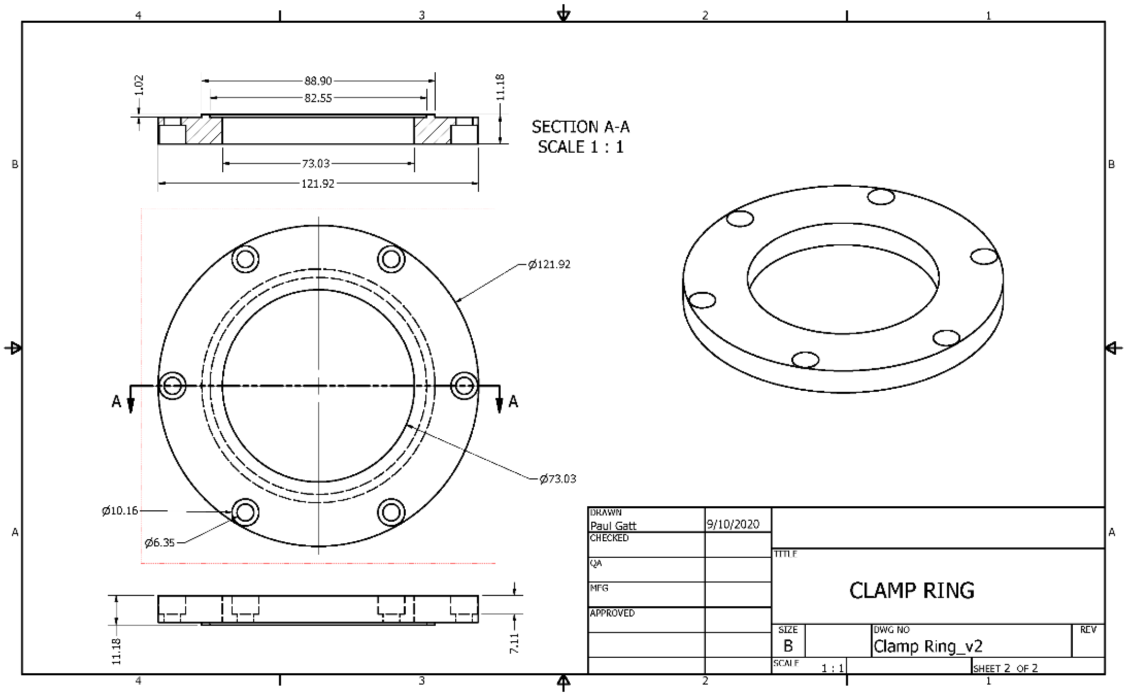

Figure S2: Schematic of the sample holder and clamp ring insert.

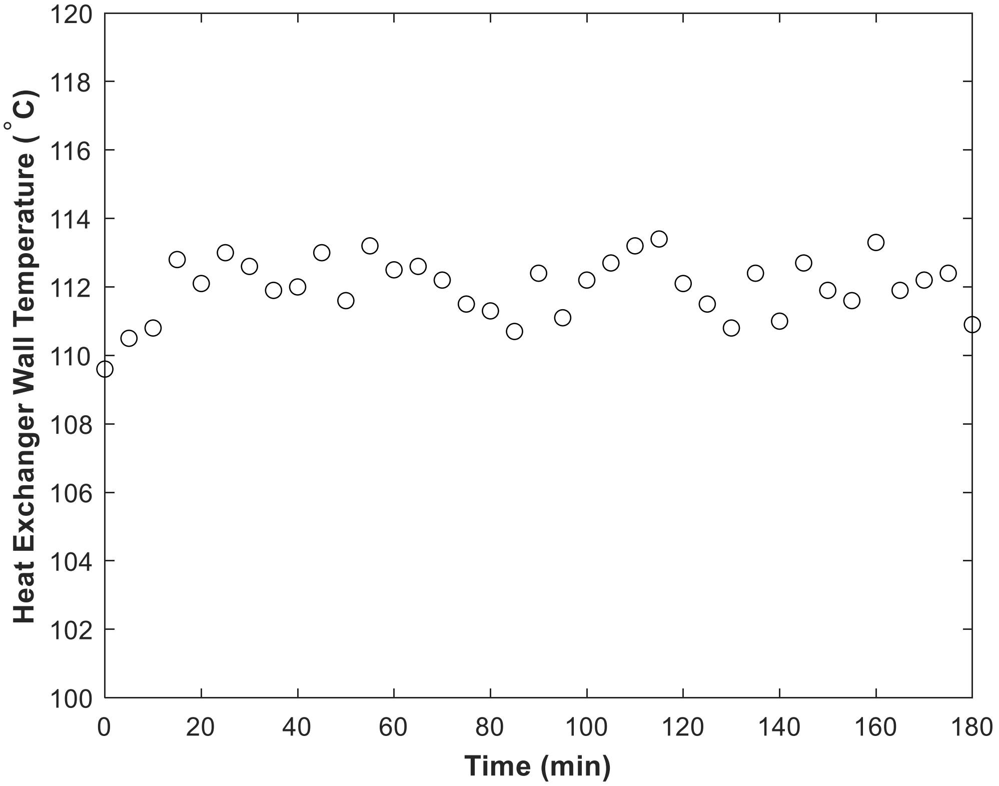

Figure S4: Plot of heat exchanger wall temperature versus time; the temperature remains consistent. The heat exchanger dries the aerosol and ensures that the concentration of aerosolized water droplets remains below 4×10^{-1} particles (0.1–0.15 µm) per cm^3 of air.

S3. Supplementary Data

Sample ID	Effective Diameter (nm)	Dispersity	Baseline Index	Count Rate (kcps)	Data Retained (%)	Diffusion Coefficient (× 10^{-8} cm^2 s^{-1})
1	129.42	0.289	7.1	635.0	100.00	3.792
2	127.49	0.299	9.9	647.8	100.00	3.849
3	126.26	0.283	6.9	641.0	100.00	3.887
4	123.33	0.291	9.3	643.5	100.00	3.970
5	120.87	0.288	7.9	639.2	98.04	4.060
6	121.66	0.283	7.6	634.3	99.67	4.030
Mean:	124.84	0.289	8.1	640.1	99.67	3.934
Std Err:	1.39	0.002	0.5	2.1	0.33	0.044
Std Dev:	3.41	0.006	1.2	5.1	0.80	0.107

Table S2: Summary of DLS data from six replicate samples of the polystyrene latex.

Table S3: Summary of zeta potential from four replicate samples of the polystyrene latex.

Sample ID	Zeta Potential (mV)	Mobility (µm s^{-1})/(V cm^{-1})	Conductance (µS)	Sample Count Rate (kcps)	Ref. Count Rate (kcps)
1	-58.40	-4.56	1,142	484	1,067
2	-56.33	-4.40	1,142	484	1,067
3	-53.56	-4.18	1,142	484	1,067
4	-52.09	-4.07	1,142	484	1,067
Mean:	-55.10	-4.31	1,142	484	1,067
Std Err:	1.41	0.11	0	0	0
Std Dev:	2.82	0.22	0	0	0
